# Fear of COVID-19 and Stress-Relieving Practices Among Social Media Users, Makkah Region, Saudi Arabia

**DOI:** 10.7759/cureus.15817

**Published:** 2021-06-21

**Authors:** Ehab A Abo-Ali, Ahmed H Mousa, Mentulla W Omar, Shayma S Al-Rubaki, Wessam A Ghareeb, Sameh Zaytoun

**Affiliations:** 1 Community Medicine Department, Faculty of Medicine, University of Tanta, Tanta, EGY; 2 Medicine and Surgery, Batterjee Medical College, Jeddah, SAU; 3 Neuro-Psychiatry Department, Faculty of Medicine, University of Tanta, Tanta, EGY; 4 Department of Community Medicine, Faculty of Medicine, South Valley University, Qena, EGY

**Keywords:** fear of covid-19, adults, saudi arabia, covid-19 outbreak, stress-relieving

## Abstract

Background

The ongoing COVID-19 pandemic and its associated consequences can trigger feelings of fear, concern, and anxiety among the population, leading to unfavorable consequences on mental health. This study aimed to assess fear of COVID-19 and stress-relieving practices among social media users in the Makkah region, Saudi Arabia.

Methods

A cross-sectional analytic study was conducted among 532 adults inhabiting the Makkah region of Saudi Arabia over a period of one month, from June 15 to July 15, 2020. A predesigned, self-administered questionnaire, including assessments of fear of COVID-19 and stress-relieving practices, was used for data collection.

Results

The mean Fear of COVID-19 Scale score was 17.3±5.21 out of 35. Individuals aged 30-49 years and married individuals had higher mean scores (18.4±5.20 and 18.4±5.29, respectively) compared to other groups (p<0.05). Additionally, individuals with histories of anxiety and depression, individuals suffering from chronic diseases, and those who did not exercise regularly had higher levels of fear compared to other groups (p<0.05). Practicing religious and spiritual rituals was the most commonly adopted stress-relieving practice among study participants (68.6%).

Conclusion

Adults in Saudi Arabia have considerable levels of fear of COVID-19. Special attention is recommended for highly susceptible groups. Additionally, mental health education programs are recommended for the promotion of the community’s psychological resilience in such a global crisis. Spiritual aspects should be included in such mental health education programs.

## Introduction

The emergence of the novel coronavirus disease 2019 (COVID-19), caused by the severe acute respiratory syndrome coronavirus 2 (SARS-CoV-2) and first reported in December 2019 in the city of Wuhan of Hubei Province in China, has been a major point of global concern. The World Health Organization declared the COVID-19 outbreak a public health emergency of international concern on January 30, 2020, and a global pandemic on March 11, 2020 [1,2]. As of the time of writing this article (November 8, 2020), more than 49 million cases of COVID-19 have been confirmed in over 200 countries, with the number of confirmed deaths approaching 1.5 million [3].

In Saudi Arabia, the number of confirmed cases of COVID-19 is 350,229, with 5,525 deaths and 336,966 recoveries. This represents a low mortality rate of 1.58% and an impressive recovery rate of 96.21% as of November 8, 2020 [4,5]. In line with international infection control guidelines, the government of Saudi Arabia promptly implemented necessary preventive measures in workplaces, restaurants, mosques, markets, and community gatherings across the country, including social distancing, wearing masks, practicing hand hygiene, lockdowns, travel restrictions, voluntary self-quarantine, online education, and working from home [6].

The ongoing COVID-19 pandemic and its associated consequences can trigger feelings of fear, concern, and anxiety among the population. A study including 1,304 participants from 75 cities in Turkey found a strong positive association between the pandemic and fear among the population, leading to unfavorable consequences such as depression, anxiety, psychological distress, and poor mental health [1]. A new term, “coronaphobia,” is being used to delineate fear of COVID-19 [7]. A systematic review of 19 studies from eight countries described the high rates of anxiety, depression, post-traumatic stress disorder, psychological distress, and stress among people during the COVID-19 pandemic [8]. Repercussions of extreme fear can include issues such as social withdrawal, stigmatization, and social discrimination and can also end with suicide, as with cases reported in India and Bangladesh [1,7,9-11]. Another study found a positive impact of wearing face masks on the physical and mental health of the population in comparison with a community that discouraged face masks [12]. Populations vulnerable to increased levels of fear include the elderly, pregnant women, and patients with preexisting psychiatric illnesses [13].

In these extraordinary circumstances resulting in unusual challenges for everyone, people must adopt coping strategies against fear and stress in an attempt to maintain physical and mental stability and wellness. Higher stress levels can lead to anxiety and disturbed sleeping and eating patterns and, subsequently, worsening physical and mental health conditions [14]. Although coping strategies can vary from person to person, recommendations from the Saudi Center for Disease Prevention and Control include decreasing time spent watching or reading the news, self-educating using reliable resources, participating in self-relaxation, practicing hobbies, communicating with others, exercising, and practicing healthy eating [15].

While a multitude of ongoing studies concerning the physical manifestation, treatment, and vaccination of COVID-19 are emerging, there are relatively few studies investigating the effects of the pandemic and related situations on the mental health of the population. This study aimed to assess fear of COVID-19 and stress-relieving practices among social media users in the western region of Saudi Arabia.

## Materials and methods

Study design and setting

This was a cross-sectional, analytic study in Saudi Arabia conducted over a period of one month (June 15 to July 15, 2020).

Study population and sampling technique

All adults who were aged 18 years and above, living in Makkah Region, Saudi Arabia, and agreed to participate in the study were eligible for recruitment. The minimum sample size, calculated using a level of confidence of 95%, expected prevalence of 50%, and precision of 0.05-was found to be 384. For increased accuracy, the study’s sample size was increased to 532. To comply with the physical distancing rules in response to the COVID-19 pandemic, convenient and snowball sampling techniques were adopted for recruitment of study participants through online invitations on various social media platforms. 

Study tool

Data were obtained using a predesigned online questionnaire. The survey was available in both Arabic and English. For each participant, the following information was collected: (a) sociodemographic, occupational, and health characteristics and relevant family history; (b) Fear of COVID-19 Scale score (this was developed and validated by Ahorsu et al. [9] in March 2020, and its Arabic version was validated by Alyami et al. [16] in May 2020. This scale is composed of seven statements [e.g., “I am most afraid of Corona,” “It makes me uncomfortable to think about Corona,” “My hands become clammy when I think about Corona,” “I am afraid of losing my life because of Corona”]. For each statement, every participant was asked to express agreement on a five-point Likert scale, with “1” indicating strong disagreement and “5” indicating strong agreement. Each patient’s total score was obtained by summation of the points for all seven responses. The scale of scores ranged between 7 and 35. Higher scores indicated higher levels of fear of COVID-19); (c) stress-relieving practices (this part of the questionnaire included statements about encouraged/proposed stress-relieving practices as recommended by the CDC, such as “taking a break from news about the pandemic” and “talking with people you trust about your concerns”); the authors added an additional question about “practicing religious and spiritual rituals,” as this is expected to be of high relevance in religious communities. For each statement, the participants were asked to indicate the frequency at which they had adopted these practices in the prior month. The questioner was tested and proved to be valid and reliable. 

Ethical considerations

Approval from the Ethics and Scientific Committees (UB-RES-2020-0035) of Batterjee Medical College was obtained before initiation of the study. Informed consent was obtained online from each participant; the aim of the study was clearly explained, and selecting the "agree to participate" icon was required before proceeding to the questionnaire items. Data were collected anonymously, and the confidentiality of collected data was guaranteed. 

Data analysis

The collected data were statistically analyzed using Statistical Package for Social Sciences (SPSS) software, version 23 (IBM, Chicago, Armonk, NY). Categorical variables were presented as numbers and percentages. Numerical variables were presented as means ± standard deviations. Testing for statistically significant differences between the subgroups was carried out. Comparing mean scores between two groups and more than two groups was carried out using student’s t-test and analysis of variance (ANOVA), respectively. The level of significance was set at p<0.05.

## Results

The current study included 532 participants from the western region of Saudi Arabia. Nearly two-thirds of the participants (n=338; 63.5%) were aged between 18 and 29 years. The majority were females (n=380; 71.4%) and individuals residing in cities (n=514; 96.6%). Approximately two thirds (n=335; 63.0%) were unmarried, and more than half (n=302; 56.8%) were Saudi citizens. Over three-fourths of the respondents (n=412; 77.4%) perceived their social level as “moderate,” and the majority (n=507; 95.3%) were living with their families or flatmates. Most of the participants had a college degree or above (n=442; 83.1%) and were employed (n=426; 80.1%). More than two-thirds of study participants (n=363; 68.2%) encountered minimal social engagement in daily activities. Most of the respondents were nonsmokers (n=448; 84.2%), had no history of anxiety or depression (n=442; 83.0%), had family histories of chronic conditions (n=338; 63.5%), and did not exercise regularly (n=350; 65.8%) (Table 1).

**Table 1:** Sociodemographic, occupational and health characteristic of social media users, Western Region, Saudi Arabia (N=532)

Variables	No. (%)
Age Group	
18-29	338 (63.5)
30-49	144 (27.1)
50 and above	50 (9.4)
Gender	
Male	152 (28.6)
Female	380 (71.4)
Residence	
City	514 (96.6)
Village	18 (3.4)
Marital Status	
Married	197 (37.0)
Unmarried (single, divorced, widow...)	335 (63.0)
Nationality	
Saudi	302 (56.8)
Non Saudi	230 (43.2)
Perceived Social Level	
High	95 (17.9)
Moderate	412 (77.4)
Low	25 (4.7)
Living Arrangement	
With family or flat mates	507 (95.3)
Alone	25 (4.7)
Educational Level	
High school and below	90 (16.9)
College degree and higher	442 (83.1)
Employment	
Employed	426 (80.1)
Unemployed or retired	106 (19.9)
Nature of usual daily activities	
With minimal social engagement (remotely or office work)	363 (68.2)
With social engagement (field work)	169 (31.8)
Smoking (Current)	
Yes	84 (15.8)
No	448 (84.2)
History of Anxiety or Depression	
Yes	90 (17.0)
No	442 (83.0)
Present or family history of chronic conditions	
Yes	338 (63.5)
No	194 (36.5)
Regular exercise	
Yes	182 (34.2)
No	350 (65.8)

The highest mean fear scores were noted in response to the items “It makes me uncomfortable to think about Corona” and “I’m most afraid of Corona” (with mean fear scores of 3.3±1.08 and 3.1±0.97, respectively). The lowest mean fear scores were found in response to the items “I cannot sleep because I’m worrying about getting Corona” and “My hands become clammy when I think about Corona,” with mean scores of 1.7±0.90 and 1.6±0.87, respectively. The mean Fear of COVID-19 Scale score was 17.3±5.21 out of 35 (Table 2). 

**Table 2:** Scores of social media users' responses to Fear of COVID-19 Scale examples in western region, Saudi Arabia (n=532)

Item	Mean± SD
1. I am most afraid of Corona	3.1±0.97
2. It makes me uncomfortable to think about Corona	3.3±1.08
3. My hands become clammy when I think about Corona	1.6±0.87
4. I am afraid of losing my life because of Corona	2.6±1.18
5. When I watch news and stories about Corona on social media, I become nervous or anxious	3.0±1.09
6. I cannot sleep because I’m worrying about getting Corona.	1.7±0.90
7. My heart races or palpitates when I think about getting Corona.	2.0±1.09
Total fear of COVID-19 scale items	17.3±5.21

The mean score recorded for the middle age group (30-49 years) was 18.4±5.20, which was higher than the scores of the lower age group (18-29 years) and the older age group (≥50 years) (16.8±5.07 and 16.5±5.56, respectively). The differences between all three age groups were statistically significant (p<0.05 ). Moreover, significantly higher mean fear scores were related to marital status; married participants revealed higher mean scores than unmarried ones (18.4±5.29 and 16.7±5.06, respectively). Comparable mean fear scores were observed for male and female participants (16.8±5.36 and 17.5±5.13, respectively), with no statistically significant difference (p>0.05). No significant differences in mean fear scores were present according to residency, nationality, perceived social levels, living arrangements, and educational levels (p>0.05 for all) (Table 3). 

**Table 3:** Fear of COVID-19 scale scores among social media users, western region, Saudi Arabia, in relation to socio-demographic characteristics

Socio-demographic characteristics	Fear of COVID-19 scale scores		t-test/ANOVA p value
	No.	Mean±SD	
Age group			
18-29	338	16.8±5.07	< 0.001^*^
30-49	144	18.4±5.20	
50 and above	50	16.5±5.56	
Gender			
Male	152	16.8±5.36	0.139
Female	380	17.5±5.13	
Residence			
City	514	17.4±5.18	0.086
Village	18	15.3±5.64	
Marital status			
Married	197	18.4±5.29	< 0.001*
Unmarried	335	16.7±5.06	
Nationality			
Saudi	302	17.2±5.07	0.663
Non Saudi	230	17.4±5.38	
Perceived social level			
High	95	17.1±5.10	0.143
Moderate	412	17.2±5.05	
Low	25	19.3±7.56	
Living arrangement			
With family or flat mates	507	17.3±5.18	0.658
Alone	25	16.8±5.76	
Educational level			
High school and below	90	17.4±5.91	0.916
College degree and higher	442	17.3±5.06	

There were no statistically significant differences between mean fear scale scores with regard to employment status, nature of daily activities, or smoking habits (p>0.05 for all). However, significantly higher mean fear scores were associated with other health-related parameters. Participants with histories of anxiety or depression had significantly higher mean fear scores compared to those who did not have histories of such conditions (16.2±5.32 and 14.4±4.14, respectively). Participants with present or family histories of chronic conditions demonstrated significantly higher mean fear scores compared to those who did not suffer from or have family histories of such conditions (17.8±5.53 and 16.5±4.52, respectively; p<0.05). Additionally, participants who did not exercise regularly demonstrated higher mean fear scores than those who did exercise regularly (17.7±5.20 and 16.6±5.21, respectively; p<0.05) (Table 4).

**Table 4:** Fear of COVID-19 scale score among social media users, western region, Saudi Arabia, in relation to occupational and health characteristics (n=532)

Occupational and health characteristics	Fear of COVID-19 scale scores		t-test/ANOVA p-value
	No.	Mean±SD	
Employment			
Employed	426	17.2±5.17	0.440
Unemployed or retired	106	17.7±5.37	
Nature of usual daily activities			
With minimal social engagement (remotely or office work)	363	17.5±5.11	0.389
With social engagement (field work)	169	17.0±5.44	
Smoking (Current)			
Yes	84	18.1±5.03	0.113
No	448	17.1±5.23	
History of anxiety or depression			
Yes	90	16.2±5.32	< 0.001*
No	442	14.4±4.14	
Present or family history of chronic conditions			
Yes	338	17.8±5.53	0.005*
No	194	16.5±4.52	
Regular exercise			
Yes	182	16.6±5.21	0.031*
No	350	17.7±5.20	

The majority of study participants (n=365; 68.6%) stated that their most common method of coping with stress was “practicing religious and spiritual rituals.” “Self-care” was found to be the second most commonly practiced stress-relieving strategy (n=285; 53.6%). Conversely, the least-practiced coping strategy was found to be “connection with others” (n=180; 34%) (Table 5).

**Table 5:** Stress-coping practices in response to fear of COVID-19 among social media users, western region, Saudi Arabia (n= 532)

Stress-coping practices	Study participants’ responses (n= 532)						Rank
	Usually		Sometimes		Never		
	No.	%	No.	%	No.	%	
Takes breaks from watching, reading, or listening to news stories, including social media about the pandemic	223	41.9	243	45.7	66	12.4	4
Taking care of your body by meditation /healthy eating habits /regular exercise & sleep /avoiding alcohol & drugs	285	53.6	204	38.3	43	8.1	2
Practicing religious and spiritual rituals	365	68.6	126	23.7	41	7.7	1
Makes time to unwind (try to do some other activities you enjoy)	278	52.2	232	43.6	22	4.1	3
Connect with others (talk with people you trust about your concerns and how you are feeling)	180	34.0	264	50.0	88	16.0	5

## Discussion

The COVID-19 pandemic has placed an overwhelming burden on healthcare systems and economies around the world. This global pandemic can provoke feelings of fear and concern among the population due to the highly contagious nature of the virus, its associated health complications, and the economic and psychological repercussions of the preventative measures imposed around the world [1]. 

In this study, the majority of survey respondents were females. This female response dominance may be explained by the fact that females are generally more motivated to participate in online surveys.This is in accordance with similar studies in Turkey and Eastern Europe [1,17]. However, marginally male-dominant responses were found in other studies [9,16]. Although female respondents exhibited higher levels of fear than male respondents, this difference was not significant. On the other hand, multiple studies in Eastern Europe, Israel, and the USA declared that females had higher levels of fear of COVID-19 [17-19]. Interestingly, respondents aged between 30 and 49 years revealed significantly higher levels of fear than younger and older age groups. This could be explained by a higher sense of responsibility in this stage of life. Furthermore, a study by Leo and Trabucchi revealed heightened levels of fear among Italian senior citizens, intensified by feelings of isolation, loneliness, and loss of hope due to multiple preexisting chronic diseases, demoralization, and social negligence [20]. However, in line with religious beliefs and traditions in Saudi Arabia, younger family members take care of the financial and health requirements of the elderly. This leads to feelings of security and serenity despite the high risk of health complications among the elderly. The present study found a remarkable association between married individuals and increased fear of COVID-19. This is in agreement with a study by Fitzpatrick et al., potentially indicating increased concern for the health and well-being of loved ones in this population [19].

No significant relationship was found between fear and social level or nationality. This can be attributed to the Saudi governmental efforts in treating all COVID-19 patients equally and free of charge, irrespective of their nationality or social level [21]. Meanwhile, a study in the USA showed increased fear among individuals of certain races and citizens born in other countries. Additionally, another study in Israel revealed a higher level of fear among individuals of low socioeconomic levels [18,19]. Additionally, living arrangements seemed to have no substantial relationship with fear of COVID-19; people living with family may have a sense of unity and security, while individuals who live alone might also feel safe due to the decreased risk of infecting family members. Furthermore, no evident association was found between educational level and fear of COVID-19. Among people with lower education, there is a lower level of proper knowledge about the outbreak and its effects, leading to a false sense of safety. Conversely, people with higher education are more likely to be familiar with the relevant evolving information and safety protocols, leading to a sense of safety.

The current study revealed no significant association between fear of COVID-19 and employment status. This is in agreement with the results of a similar study conducted in China [22]. One potential explanation is that employees who returned to work after the reduction of lockdown restrictions were aware of safety precautions, which enhanced their psychological resilience. Various preventive measures were announced to the population (e.g., wearing face masks and frequently sanitizing hands) [23]. Furthermore, unemployed or retired individuals showed nearly the same level of fear of COVID-19 since they are expected to experience lower levels of social interaction during the pandemic. Similarly, no association was found between fear of COVID-19 and the nature of practiced daily activities, either with minimal or evident social engagement. Though smoking is a factor highly associated with complications from COVID-19, the present study showed no association between fear of COVID-19 and smoking habits. This finding is in line with results from a previous study conducted in Vietnam, which revealed that smoking was a stress reliever among medical students, especially during stressful times [24].

The present study showed a significant association between fear of COVID-19 and history of anxiety and depression. Similarly, a previous study in Brazil showed that anxiety and depression can affect individuals who are most likely exposed to high rates of stress and are on the frontlines with respect to pathogens, such as individuals in healthcare professions [25]. Additionally, other studies conducted in China revealed high rates of stress symptoms and anxiety among doctors and nurses [26,27]. 

The current study demonstrated a significant relationship between fear of COVID-19 and associated chronic illnesses. This result is supported by a study previously conducted in Turkey [28]. This may be anticipated, especially with the emphasis of World Health Organization, that individuals who suffer from chronic illnesses are more likely to contract COVID-19 with higher mortality rates [28]. In addition, patients who are frequently treated with immunosuppressive agents and cytotoxic drugs tend to be immunocompromised and more susceptible to infections. People with systemic lupus erythematosus are considered a vulnerable population for SARS-CoV-2 infections and COVID-19. Both systemic lupus erythematosus and COVID-19 have been shown to manifest multi-organ complications, such as interstitial pneumonia, cytopenia, arthralgia, myocarditis, and hemophagocytic lymphohistiocytosis [1,29]. 

Additionally, the present study revealed a significant association between lack of regular exercise and increased fear of COVID-19. Frequent physical exercise is associated with the maintenance of good physical and mental health and is considered an effective mechanism of coping with stress [7].

The present study revealed that “practicing religious and spiritual rituals” was the most frequently adopted stress-relieving practice among adults in Saudi Arabia. This finding is not surprising since the study was conducted in a mostly religious community. Conversely, a study conducted in the USA revealed that the population has directed their thoughts to extreme pessimism and hopelessness, with more suicidal ideations, alcohol, and drug abuse during the COVID-19 crisis [2]. Moreover, another significant strategy for coping with stress during the pandemic is taking care of the body by way of meditation, healthy eating habits, regular exercise and sleep, and avoidance of alcohol and drugs. This is supported by another study in India that showed how such strategies can promote the mental health of individuals during these stressful times [3]. At the very least, stress-relieving practices included staying in touch with friends and family members using online platforms and social media.

Strengths and limitations

This study has the strength of being the first to explore and assess the fear of COVID-19 and stress-relieving practices in the community of Saudi Arabia. The non-probability sampling technique is a limitation of the study but was adopted due to the hindering effect of lockdown during the study period. The bias of this non-probability sample was reduced by enlarging the sample size. Additionally, the study only recruited subjects who had access to social media platforms. 

## Conclusions

Considering the results of the present study, we can conclude that adults in Saudi Arabia have been experiencing fear of COVID-19 to a considerable degree. Particular groups, such as middle-aged, married, those with a history of anxiety and depression, subjects suffering from (or with a family history of) chronic diseases, and persons neglecting regular exercise reported higher levels of fear. The most commonly adopted stress-relieving practice among the study participants was “practicing religious and spiritual rituals”. Special attention is recommended for the care of the aforementioned susceptible groups, and mental health education programs should be initiated for the promotion of the psychological resilience of the community. Spiritual aspects should be included in the construction of such health education programs. A population-based study is recommended for addressing the magnitude of the problem on a wider spectrum. 
